# Artificial Turf: Contested Terrains for Precautionary Public Health with Particular Reference to Europe?

**DOI:** 10.3390/ijerph14091050

**Published:** 2017-09-12

**Authors:** Andrew Watterson

**Affiliations:** Occupational and Environmental Health Research Group, Centre for Public Health and Population Health Research, University of Stirling, Scotland FK9 4LA, UK; aew1@stir.ac.uk; Tel.: +44-017-8646-6283

**Keywords:** artificial synthetic pitches, play surfaces, public health, crumb rubber, tires

## Abstract

Millions of adults, children and teenagers use artificial sports pitches and playgrounds globally. Pitches are artificial grass and bases may be made up of crumb rubber from recycled tires or new rubber and sand. Player injury on pitches was a major concern. Now, debates about health focus on possible exposure and uptake of chemicals within pitch and base materials. Research has looked at potential risks to users from hazardous substances such as metals, volatile organic compounds, polycyclic aromatic hydrocarbons including benzo (a) (e) pyrenes and phthalates: some are carcinogens and others may be endocrine disruptors and have developmental reproductive effects. Small environmental monitoring and modelling studies, often with significant data gaps about exposure, range of substances monitored, occupational exposures, types of surfaces monitored and study length across seasons, indicated little risk to sports people and children but some risk to installation workers. A few, again often small, studies indicated potentially harmful human effects relating to skin, respiration and cancers. Only one widely cited biomonitoring study has been done and no rigorous cancer epidemiological studies exist. Unravelling exposures and uptake over decades may prove complex. European regulators have strengthened controls over crumb rubber chemicals, set different standards for toys and crumb rubber pitches. Bigger US studies now underway attempting to fill some of the data gaps will report between 2017 and 2019. Public health professionals in the meantime may draw on established principles to support greater caution in setting crumb rubber exposure limits and controls.

## 1. Background

Artificial surfaces and turf, sometimes called “synthetic” surfaces, first appeared on a major professional sporting pitch as an “innovative” substitute for natural grass in the USA in 1966 and in Europe in the 1980s. They are marketed across North America, Asia, Africa, Australia and South America in many forms and are now in playgrounds, amateur pitches and even in domestic gardens. It is a large market. The technology and materials used are evolving globally. Hence, it can be difficult to assess their specific or combined health effects, if any, or identify exactly what crumb rubber materials and what quantities and from what source have been or are used now in the original installations in very varied locations and climates and with what chemical and metal concentrations. For the same reason, it is difficult to compare environmental monitoring studies of crumb rubber that have been conducted in many different settings. Nevertheless, across the world, hundreds of thousands of adults and children come into contact with these surfaces each week, yet remarkably little is known about their exact health risks or benefits especially over the long term. In contrast, there have been hundreds of sports injury studies carried out for artificial pitches.

The global use of artificial sports and play surfaces has brought claimed benefits from exercise, perhaps cost-savings and greater access in all weathers along with claimed major chemical exposure risks [1]. Early problems with sports injuries on these pitches led to changes in the materials used but relatively little attention was paid to any health risks from the surfaces. A “rapid” review of the toxicological and exposure literature on artificial pitches and crumb rubber use in such pitches reveals a very patchy picture, with no fully conclusive assessments of either risks or acknowledged hazards. The US EPA in 2016 brought together an extensive assessment of both knowledge and data gaps on crumb rubber used on playing fields on playgrounds when it prepared its federal action plan [2]. This drew on important US EPA reports from 2009 and 2015 that explored the field. Their findings are flagged in later sections. Some of the key studies that identify lack of risks from crumb rubber hazards, data gaps and potential risks are listed in tables below. The grey literature provides a host of conflicting claims from opposing sides in the debate ranging from pitches have caused or may cause cancers like Hodgkin’s lymphoma and leukaemia, respiratory problems and burns to all pitches are safe and crumb rubber presents no risks to users, workers or the environment [3,4,5,6,7,8,9]. However, many data gaps remain with regard to potential exposures and effects of materials singly and in various combinations. Toxicology too has moved on since the installation of the first pitches with evidence emerging for example about very low level chemical exposures to endocrine disruptors, sometimes present in and around artificial surfaces and their potential adverse effects. How such gaps are now interpreted and how existing research findings are weighted and used is also debated primarily between environmental groups, parent groups, pitch and playground purchasers and the manufacturing and supply industry. The scientific and public health communities continue to research exposures but have not yet fully established evidence of absence of risk from known hazards with artificial turf and infills.

This paper therefore explores some of these debates and how related public health policy and practice issues are addressed. These sometimes complex conflicts may include different assessments about “natural versus synthetic” materials, old versus new technologies, cost versus environmental considerations, waste disposal versus recycling, occupational versus environmental exposures, profitable industry versus impoverished communities, professional and sporting versus infant and children, and acute versus chronic exposures. The old adage of Geoffrey Rose about the public health implications of large populations exposed to low toxic exposures being greater than small populations exposed to large doses would also appear to be especially relevant in this debate [10].

The UK’s Chief Scientific Advisor’s Report: “Innovation: Managing Risk, Not Avoiding It” presented a dominant paradigm in 2014 about risks, hazards and their management and in a number of ways echoed policies about the need to reduce risk aversion. Within such an approach is embedded a view that absence of evidence on risks from established hazards can be interpreted as evidence of absence [11]. Where no alternatives are available and where public health benefits are considerable and adverse effects are likely to be minimal, such an approach may merit consideration. Where there are alternatives, where risks may be unknown or known but not fully quantified and could be high for large numbers of people or vulnerable populations, the approach is deeply flawed.

Some consumers, users and researchers advocate the opposite view to the UK’s Chief Scientific Advisor and argue society and regulators should look for evidence of a lack of serious risks to health when large and sometimes vulnerable populations, at critical points in their growth or development, may be exposed to processes and substances before introducing or continuing with new technologies and materials. This is especially so if the public health benefits may be small or can be provided by alternative less hazardous materials or processes and the data gaps are significant.

## 2. Scale of Usage and Possible Exposures to Chemicals and Metals

First generation grass was made from nylon or polypropylene. Second generation pitches had sand infills as bases. Later pitches and play surfaces, third generation and beyond, which may vary significantly with regard to constituents used depending on geographical locations and climate, contained various combinations of materials including shock pad (which may also be made of crumb rubber), natural products such as cork or coir, a coconut-derived material, sand and crumb rubber as “soil” or “infills” not grass substitutes [12]. The crumb rubber often but not always comes from recycled vehicle tires and this might seem to be a safe waste disposal strategy. The crumb may also come from more expensive virgin rubber such as thermoplastic elastomers, TP and EPDM made from ethylene, propylene and diene. Beneath the crumb could be sand as ballast [13]. Between 20,000 and 40,000 tires may be used to create crumb rubber infill for an average football pitch [14]. Crumb rubber and other materials are also used in various forms in rubber mulch and mats in children’s playgrounds. In the USA, around 17.5 million tires were used to cover children’s playgrounds [15]. Third generation (3G) pitches have been built across the world for baseball, American football, football, rugby, hockey as well as on athletics tracks for professional and amateur players. Fourth generation artificial AstroTurf combines sand and rubber infill and 5G and 6G pitches are being developed again with crumb rubber. USA has an estimated 12,000 artificial turf pitches made from around 10 million tires [15]. In Great Britain, 605 3G pitches were registered just with the Football Association in 2015/2016 including ones in schools and recreation grounds [16]. In Europe, each year 3.4 million tons of end of life tires are collected and of these 1.3 million tons are processed for recycling by shredding and granulation with crumb rubber for rubber infill or synthetic turf being a major application [17].

Crumb rubber from tires may contain for example various metals such as cadmium, chromium, zinc, aluminium and lead; oils containing various chemicals including volatile organic compounds (VOCs) and polycyclic aromatic hydrocarbons (PAHs) such as benzo (a and e) pyrenes, benzo (a) anthracene as well as phthalates. Several of these are known or suspect carcinogens, neurotoxins and potential endocrine disruptors. Some have been detected at very low levels from pitch samples. Hence, understanding effects of any possible health effects as well as uptake by inhalation, skin absorption or ingestion is critical. Carbon nanotubes too may be present in or by-products of artificial grass and crumb rubber manufacture and use [12,13,14].

## 3. The Debate about Hazards and Risks of Crumb Rubber

There are a number of competing interests between manufacturers, suppliers, purchasers, parents and users with regard to artificial pitches and playgrounds and their effective oversight by governments and health, safety and environment agencies. Debates about various pitches have covered hazards and risks, waste disposal and recycling and sustainability strategies, exposures and uptake, costs and benefits linked to alterative pitch options, externalizing of costs of any potential adverse human effects from manufacturers onto communities and the health services, chemical control limits, health surveillance. Researchers, through a range of funders including local and central government, charities and industry, have investigated aspects of theoretical and field exposure of users and also communities where crumb rubber leaching into water was assessed.

Artificial turf manufacturers claim their products are safe and superior to natural grass [12,13]. The industry argues for a balance between economic and environmental goals linked to long term solutions on the sustainable use of “secondary raw materials”. Natural grass sales companies and “organic infill” manufacturers make similar claims for their products and may dispute artificial turf industry claims [18,19]. Artificial turf manufacturers and suppliers consider their products are adequately regulated as exposures have been measured under legal limits and hence do not provide a public health threat but instead a valuable means of dealing with end of life tires that cannot be landfilled and might otherwise present a serious environmental problem. The industry cites various international studies, many but not all peer reviewed in scientific journals, from researchers and agencies in several countries to support their case although a number of these studies qualified their conclusions in terms of design limitations and on occasions lack of data. Other researchers have challenged safety standards and some found pollutants from artificial surfaces but disagree about their human health significance. Some environmental groups continue to flag public health concerns about these surfaces and advocate a precautionary public health approach based on using natural turf surfaces and organic infills instead [20]. Some parents and sports bodies remain concerned, demonstrated by sources above from the grey literature, both by the contradictory views expressed by researchers about existing data and data gaps for materials they assumed were established as “safe” over fifty years ago.

The debate about artificial pitches in Europe has been linked to chemical regulations and moves in the mid-2000s to establish EU chemical standards for pitches and surfaces [3,6]. At that time, the Dutch, recognizing potential risks, reportedly proposed the following requirements for such a standard. Firstly, “the surface layer of synthetic turf shall not contain or release any substances that are carcinogenic, mutagenic or reprotoxic; persistent, bioaccumulative and toxic; very persistent and very bioaccumulative; toxic in contact with skin, very toxic in contact with skin or may cause sensitization in contact with skin”. Secondly, “rubber in synthetic turf shall not exceed 0.85% by weight of substances that may cause cancer; may cause heritable genetic damage; may cause cancer by inhalation; are very toxic to aquatic organisms and that can cause long-term harmful effects on the aquatic environment; toxic to aquatic organisms, may cause long-term adverse effects in the aquatic environment; are harmful for aquatic organisms, may cause long-term adverse effects in the aquatic environment; may impair fertility; may cause harm to the unborn child” [21]. These requirements would not necessarily have prevented the installation of these pitches but would have raised awareness and increased the flow of information to those either purchasing or playing on them now being proposed in 2017.

Most but not all research interest has focused on crumb rubber recycled from end of life tires and, within the rubber, on those PAHs and VOCs that are known or suspect human carcinogens, mutagens and endocrine disruptors. For PAHs, benzo (a) pyrene, dibenz (a) and (h) anthracene and related chemicals have been of greatest concern. These are often the chemicals that EU regulators officially recognize and list as known or suspect carcinogens, mutagens and reproductive hazards. With the tire and rubber industry, EU agencies have been working towards reducing levels of these chemicals in crumb rubber in recent years. They are also the chemicals over which there has the greatest debate between national governments, regulators and tire and rubber and rubber crumb companies.

The Registration, Evaluation, Authorisation and Restriction of Chemicals (REACH) Regulations that came into force in 2007 restricted the levels of various PAHS in new tires, retreads and the extender oils used to make them from 1 January 2010 onwards. Eight PAHS were covered: benzo (a) pyrene (BaP), Benzo (e) pyrene (BeP), benzo (a) anthracene (BaA), chrysene (CHR), benzo (b) fluoroanthene (BbFA), benzo (j) fluoroanthene (BjFA), benzo (k) fluoroanthene (BkFA) and dibenzo (a,h) anthracene (DBAhA) [22].

Entry 28 of Annex XVII to the REACH Regulation established a general limit on the presence of carcinogenic, mutagenic and toxic for reproduction substances placed on the market, or used by themselves, or in mixtures, for supply to the general public. This resulted in a maximum limit of 0.1% for some PAH substances in rubber tires and 0.01% for others (such as benzo (a) pyrene) [23].

The European PAH restriction in December 2013 in the UK setting noted that any rubber or plastic items could no longer be supplied to the general public where they came into regular direct contact with the human skin or mouth and where they contained more than 1 mg/kg of the PAHs listed above. The same restriction applied to toys and childcare items, but the PAH limit was set for these at 0.5 mg/kg [22].

The tightening of chemical control standards described here is not an unusual step: for example, asbestos, benzene, and beryllium standards have all been raised and acceptable exposure levels lowered due to later research. What would be exceptional is to come across a case where standards are loosened. Tougher standards normally reflect more recent research indicating established or potential problems and risks not recognized earlier [24]. This phenomenon explains why public health practitioners and parents often wish to adopt the precautionary principle if alternatives products are readily available and known to present evidence-based lower risks than newer products [25]. Parts of the rubber industry have recently contested interpretations of European controls on crumb rubber and in November 2015 argued crumb rubber in artificial pitches was part of the “infrastructure”, a “mixture” and not an “article” under European provisions. Hence, crumb rubber should avoid the tougher 0.5 mg/kg (0.00005% of a product) standard set on eight PAHs for toys and meet only the lower standard on mixtures [26]. However, there may be a perception by parents and users that, if crumb rubber safety margins are already so high and the product is so safe, then tightening controls to meet the European 2013 PAH Toy standard should be possible, albeit more expensive and technically demanding for industry.

Crumb rubber safety data sheets, some were still referred to as material safety data sheets when produced, available on the web and from pitch sellers and reviewed cannot resolve these debates (Supplementary Table S1). Their toxicity information, significantly, may vary between dates of installation and between manufacturers: an important consideration when pitches may be topped up with crumb rubber from different suppliers over a number of years. The source of the crumb rubber is also missing for some data sheets and questions of provenance of materials in the international trade of crumb rubber have also arisen. Experiential knowledge from sports and playground staff, players and parents indicates contact with the crumb rubber through possible ingestion, skin and inhalation routes could potentially be large [27,28]. Data on physical and chemical properties in such sheets can be sparse on occasions with some entries stating “no data available on the product”. Other data sheets provide details on first aid measures for inhalation and ingestion of crumb rubber. A few sheets included details on chronic exposure and fuller information on first aid measures. The information provided acknowledges the presence of a range of hazards and potential risks in the materials with different exposures and risks for those in manufacturing and leisure use and sports maintenance. A number of “information” sheets but not full data sheets examined related to pitch and play surfaces may also be available to sports staff. These focused almost exclusively on maintenance and did not address health and safety issues.

## 4. Incomplete Knowledge

Through the 2000s, concerns about possible human health risks from chemical exposures via skin absorption, ingestion and inhalation and through leaching of chemicals into water courses increased [28,29,30,31,32,33,34,35,36]. Recent scientific studies, several of which were small scale in terms of numbers of people involved, number of sites and surfaces included, length of exposure assessments and so on did not fully measure and evaluate all the hazards and related risks posed by several generations of pitches and play surfaces [35,36,37,38,39,40,41,42,43]. Some studies focused only on exposures from outdoor pitches and not indoor ones. Some studies looked at off-gassing and inhalation but not ingestion and skin absorption. Others explored a limited number of surfaces and for a limited amount of time. Some studies identified some leachates from crumb rubber pitches but not others and again not over longer time periods. Parent groups and the media have linked the incidence of cancers such as Hodgkin’s lymphoma especially in young goalkeepers with high pitch use and contact with artificial playing surfaces [44]. However, detailed studies are lacking about denominators for reported cases, the total number of pitch users, the prevalence of the disease in the population at large and for various age groups, the type of pitch, the period of time players used pitches and longer term follow up.

## 5. Studies Indicating Probable Safety for Users and Producers

A series of particular types of exposure and scenario studies and one small bio-monitoring study found no cause for concern for players on artificial pitches that involved crumb rubber and other materials. The larger or more influential and most cited of these studies are briefly described below.

Two Norwegians Agencies in 2005/2006 looked at dermal and inhalation risks for adults and children from recycled crumb rubber and organic chemicals on pitches concluding health risks were unlikely [35,36]. The exposure study looked at three halls: one with recently laid rubber granulate (SBR rubber or Styrene Butadiene Rubber), one with granulate in use for one year and one with granulate made from thermoplastic elastomer. This indoor pitch air pollution study then informed the inhalation and oral exposure scenarios developed by the Norwegian Institute of Public Health a year later [45].

French lab-based tests, partly drawing on a pilot scenario as well as an in situ pitch test with an 11 months monitoring period, reported in 2007, were conducted by a research institute for a global artificial turf company were used to carry out both an environmental and health assessment of French materials used in 3G type-turf made from virgin rubber and recycled tires. The tests took into account ballast sand, glues and assembly bands. This research, which also draws on health risk assessments produced by other French governmental and research agencies, found levels of chemicals such as VOCs and formaldehyde were either very or relatively low with levels highest and were not considered a health threat to sports people and spectators exposed to crumb rubber. The biggest risks identified in France were to workers laying turf over a five-year period in small and poorly ventilated buildings [37].

In 2010, the Connecticut Department of Public Health carried out a conservative human health risk assessment based on air sampling of only one indoor pitch with no active ventilation and four outdoor artificial turf pitches in summer conditions. Twenty-seven chemicals of potential concern were found above background levels that could be related to pitches indoors and outdoors. The study risk assessments found “calculated risks are well within typical risk levels in the community from ambient pollution sources and are below target risks associated with many air toxics regulatory” [38]. The study team also suggested the above background levels for benzene could have been due to personal sampling equipment problems rather than indications of field-related exposure. However, the study team did not repeat the monitoring to check their hypothesis [38]. They concluded “the use of outdoor and indoor artificial turf fields is not associated with elevated health risks” [38]. The larger 2016 USEPA status report details how some of these gaps and shortcomings will be addressed [2].

An extensive 2013 review by Chinese and American researchers examined both environmental and health impacts of artificial turf. Products in artificial grass as well as crumb rubber could cause contamination and leaching. The researchers noted maintenance and low water consumption benefits of artificial turf and the capacity of natural turf to biodegrade various airborne pollutants, provide a richer ecosystem and have a negative carbon footprint. The authors’ limited small life cycle analysis of three representative artificial turf pitches and equivalent natural grass fields found the environmental impacts of natural grass were much less for 10 indicators, marginally higher on land use and significantly higher on resource consumption. With greater use of artificial pitches, it was estimated the figures would move in favour of the artificial pitches. They highlighted the fact that “many risk assessment studies” having been conducted to look at artificial turf exposure routes “with the results consistently showing that no significant health risk was associated with being on or playing on such fields” [39].

Their conclusion, based on exposure not epidemiology studies, “suggested the users of artificial turf, even professional athletes, were not exposed to elevated risks” but added caveats [39]. These included the need for more work on tire rubber degradation under field conditions, hazard substance leaching dynamics particularly as heavy metals do not degrade, and disposal and recycling of artificial pitches. The decisions by American and European Agencies to do more work on exposures would suggest not all researchers now necessarily share this paper’s conclusions especially in the light of the complete absence of epidemiology studies. No epidemiological studies of artificial pitch and surface users from the 1960s, 1970s, 1980s and 1990s are yet underway. There are also no substantial published occupational health studies of crumb rubber and artificial turf workers bar one in Taiwan.

A 2014 US study using artificial bio fluids looked at eight artificial fibres, eight infills and samples from seven fields, and found chemical exposures often below detection levels and metals at levels of “low” estimated human risks with the possible exception of lead [40].

One very small and partially industry funded 2009 Dutch study using bio-monitoring looked at seven players—over three days aged twenty or more, players training and playing one match—and found no or minimal exposure to certain chemicals from the crumb rubber pitch [41].

Health Impact Assessments from Toronto have recently compared artificial and natural turf surfaces [1] finding artificial pitches created urban heat islands and related heat illnesses along with air, dust and water contaminants and leachates. However, adverse health effects were assessed as unlikely if good installation and hygiene practices were used. Storm water run-off could be significant and artificial pitches, unlike natural grass pitches which were carbon sinks, increased greenhouse gas releases. Impacts on the built environment and communities were unclear cut but artificial pitches could replace natural green spaces, increase traffic, lighting, parking and noise in some inner city residential areas. Artificial surfaces increasing recreational use and physical activity especially in low income areas as well as providing better access for those with disabilities through all-weather use and materials. Pesticides applied to natural grass pitches are not needed on artificial pitches although other chemicals may be required to control bacteria and moulds on artificial surfaces. Risks initially identified related to abrasions and musculo-skeletal and joint injuries and these appear to have been reduced over the years with new designs and materials.

The Canadian health impact assessment from 2015 looked at toxicological risks for artificial turf based on selected government agency/state reports from the USA, Europe and Australia. Excluding reports on lead, the review examined nine such reports and eight estimated risks as low and unlikely while one from Sweden assessed risks as low but called for a ban on the use of recycled crumb rubber from tires in new artificial pitches [1]. So several studies could be interpreted as both indicating safety of such pitches and their materials as well as their hazards and potential risks [46,47,48,49,50,51,52,53,54,55] (See Table 1 below).

## 6. Studies Indicating Potential Health Problems for Pitch and Playground Users and Workers in Manufacture

Whilst some studies indicated no risks, some studies showed risks and potential health problems but assessed those risks as low. However, a number of Southern European studies on exposure, unlike those in Northern Europe, flagged serious concerns about exposure limits being exceeded for a range of chemicals present in crumb rubber and one crumb production worker study identified possible risks [46].

A 2003 occupational hygiene study of two scrap tire shredding plants in Taiwan found low levels of VOCs in the air and the presence of amines, aniline and benzothiazole which could break down into carcinogens [45]. 2-mercaptobenzothiazole is an International Agency for Research on Cancer 2A carcinogen with limited human evidence on bladder cancer and sufficient animal evidence on leukaemia. It has been “found as an environmental contaminant, and detected in crumb rubber used on playgrounds” [47].

A Swedish study in 2006 was unequivocal and stated, whilst recognizing there were gaps in knowledge that based on reviews of the literature “tyres contain several substances that are substances of very high concern. These substances may persist in the environment, they may be bioaccumulative, carcinogenic, ‘reprotoxic’ or mutagenic. This is true of, for example PAHs, phthalates and certain metals. These substances should not be released into the environment and thus waste tires should not be used for synthetic turf surfaces” [21]. However, it believed the health risks to players were “probably low” and although effects on environments would occur, they too were expected to be low.

The 2010 Connecticut study discussed above [38] has generally been viewed as giving crumb rubber the all clear but it was also open to other interpretations. On-field and background sampling found 27 chemicals of potential concern above background levels although the sampling did not involve new crumb rubber nor was necessarily done on days when off gassing of chemicals was likely to be at a maximum. The risk assessments conducted considered both play activity and children’s greater ventilation rates when compared with adults. These found cancer risks slightly above USA “de minimis” levels for all the scenarios in the risk assessments. The Connecticut report listed benzothiazole as a possible human carcinogen but with very low exposures in their study.

Spanish researchers in 2013 analysed 21 rubber mulch and seven commercial samples from playgrounds and pavers that were made up of recycled rubber tire tiles in urban areas. They did not examine sports pitches. The strengths and limitations of the study were not detailed. Thirty-one target compounds were examined including 16 PAHs as well as vulcanization additives, antioxidants and plasticizers. Extremely high levels of PAHs were recorded in the seven commercial samples from the pavers reaching values up to 1%. Phthalate plasticizers were found in all the samples analysed with pavers again recording the highest levels. The researchers concluded the “uses of recycled rubber tires, especially those targeting play areas and other facilities for children, should be a matter of regulatory concern” [48]. Careful control, restrictions on use and possibly even prohibition were mooted.

In Italy, a study of 13 artificial pitches based on crumb rubber was conducted and air sample analyses for 25 metals and nine PAHs as well as other chemicals were collected. Both static and personal samplers were used to measure pollutants and their inhalation by players and efforts were made to cover the worst case scenarios. The researchers acknowledged they had a limited dataset and viewed their findings as a preliminary study. The results showed zinc and PAH levels significantly exceeded Italian “green area” soil limits although player risks were estimated to be low [21]. The authors were aware that risk assessments were affected by numerous variables including the types of rubber involved, their chemical composition, the state of the playing fields, their age, meteorological and climatic conditions. This study raises the important issue addressed in earlier research in Scandinavia [16,17,18] and the USA but not fully resolved about metals and chemicals that may occur naturally in urban and indeed rural soils, or be transported to the soil from sources other than crumb rubber as well as crumb rubber sources. It will be important to identify all the different sources if possible. However, the total load from all sources including pitch and playground crumb rubber will be critical in public health assessments. The US EPA report [2] identified a number of studies including ones from the Californian Office of Environmental Health Hazard Assessment [33] that took soils from different samples allowing some comparisons to be made. Rhodes in 2007 and Aoki in 2008 explored metal leaching from artificial turf [49,50].

Another very small and preliminary Italian study in 2014 began to explore the hazards and risks presented by artificial turf fields based on nine crumb rubber samples and analysis of heavy metals and PAHs. Their focus was on inhalation by athletes in training and particular attention was paid to pitch surface temperatures. They found there were no EU guidelines defining measures to protect the environment and human health in relation to Styrene Butadiene Recycled Rubber in synthetic turf, only German ones, also adopted by Italian sporting bodies for some heavy metals but not PAHs [43]. The preliminary study found a range of potential risks from inhalation, absorption and ingestion with PAHs released continuously from crumb rubber due to evaporation. Several cadmium samples taken exceeded limits set by the Italian National Amateur League but levels were similar to those recorded in public parks and residential land. Very high levels of total PAHs were recorded in three samples. They concluded “although synthetic turf offers various advantages over natural grass, the quantity of toxic substances it releases when heated does not make it safe for public health” [43]. Hence a series of scientific papers, reports and policy documents flag questions about pitch and crumb rubber safety [56,57,58,59,60,61,62,63,64]. (See Table 2 below)

## 7. Regulatory and Research Agency Actions and Regulatory Standards in Europe

Health monitoring and inspection of artificial turf manufacturers, suppliers, installers of outside and indoor pitches and playground sites by environmental health or occupational health and safety agencies and researchers appears to have been limited if not non-existent in several European countries. This is remarkable because of the past history of occupational ill-health in rubber manufacture, the risks attached to the use of solvents and adhesives and the off-gassing problems created in the past by a range of industries.

The Swedish Chemical Inspectorate (KEMI) status report in 2006 found major gaps in knowledge on the subject especially relating to the release of hazardous substances from the recycled rubber tires and considered the industry should be responsible for investigating and assessing any risks. They concluded the information available at that time made reliable health risk assessment difficult because results had only come from “few measurements in few halls on few occasions and it is thus most appropriate to regard these as random samples rather than the basis of generally applicable assessments of risk” [21]. At one level, it is clear the industry should assess its health risks and cover the costs and time of staff employed to carry out those assessments. At another level there is substantial concern about what has sometimes been translated into self-regulation and self-inspection as well as self-assessment with potentially no independent oversight of the data generated by industry, the assessments made and the production, installation and maintenance practices used on artificial pitches and playgrounds. This leads in to a related debate about “better regulation, soft regulation, smart regulation”, red tape and deregulation that should be factored in to how the crumb rubber and artificial pitch industries are overseen.

The Norwegian Institute of Public Health health risk assessment for football players used scenarios to assess exposures concluded, based on knowledge in 2006, that health risks were generally very low. However, it noted data on oral rubber intake were lacking, many of the VOCs monitored were not classified so health risks were unknown, and many VOCs only had acute and not chronic effect classifications [36].

The European Commission and its EU member states have accepted there is a need to assess whether rubber crumb used as infill in sports pitches poses a human health risk. The Commission tasked the European Chemicals Agency (ECHA) to carry out such an evaluation and ECHA is working with other authorities in the EU and the US to do this. ECHA aims “to identify any hazardous substances in the recycled rubber filling that may pose a health risk such as PAHs which are already extensively restricted by EU legislation and assess the risk resulting from skin, oral and inhalation exposure to these substances in recycled rubber filling used on both open air and indoor sports grounds” [51].

The US EPA in 2016 found “Limited studies have not shown an elevated health risk from playing on fields with tire crumb, but the existing studies do not comprehensively evaluate the concerns about health risks from exposure to tire crumb”. This triggered their new research programme [2]. The gaps they found included the following: no epidemiology studies; limited studies on playgrounds, indoor fields, occupational exposures; limited information on biomonitoring and bioavailability; limited data on some organic chemicals; gaps on dermal and ingestion pathways linked to particle exposures; gasp on constituents and scenarios of highest possible exposure; gaps on cumulative aggregate assessments; studies on alternative infills and materials and natural grass fields; and other sources of exposure to chemicals found in crumb rubber. As the Agency noted “Information specific to the frequency and duration of synthetic field and playground uses, physical activities, contact rates, and hygiene are limited. Exposure factor data are not available either across the wide variety of sports and recreational users of synthetic turf fields and playgrounds with tire crumb rubber, or for occupational exposures” [52].

Within the EU, an expert group advises both the Commission and ECHA matters relating to REACH and related classification and labelling of chemicals. This is the Competent Authorities for REACH and CLP: CARACAL [53].

Perhaps surprisingly, this is only now taking place for a technology that emerged in the 1960s. In some countries, there have only been safety checks on the artificial turf manufacturing companies because of concerns about machinery. The UK regulatory agency has not, according to the above Freedom of Information request submitted in June 2016, conducted checks except on injuries and two checks on noise and fumes in the sector but no other checks on occupational health issues over a 10-year period (UK HSE Freedom of Information Request Reference No: 201605332) (Supplementary Table S2).

## 8. Conclusions

Despite the USA first pioneering artificial turf technologies, US and other countries’ regulatory agencies still cannot resolve risk levels from crumb rubber hazards. The most recent global research and reviews, whilst allaying some fears, have also lead to recommendations for tougher controls on crumb rubber chemicals in the light of data gaps and inadequately designed studies. This is consistent with long established precautionary principles. The United States Environment Protection Agency is now trying to “fill important data and knowledge gaps, characterize constituents of recycled tire crumb and identify ways in which people may be exposed to tire crumb based on their activities on the fields” [52]. Such work will continue through 2017. The Californian Environment Protection Agency’s Office of Environmental Health Hazards Assessment project too is exploring artificial turf surfaces through hazard identification, exposure scenario development, sampling and analysis of new and in-field artificial turf, and biomonitoring study protocol development and will report in 2019. The project covers indoor and outdoor surfaces looking at crumb rubber and artificial grass blade chemicals, releases from pitches of all ages and varied weather conditions, exposures possibly due to ingestion and inhalation as well as skin absorption across ages, athlete use of surfaces and potential exposure times including possible effects on individuals and sensitive populations such as children [54].

Many governments permit crumb rubber use as a means of recycling rubber tires and reducing landfill but paradoxically many governments oppose the landfilling of tires because of concerns about leachates. Regulators in Europe do not apply the Registration, Evaluation, Authorisation and Restriction of Chemicals regulations in force from 2007 to the waste tires but will do so to any new products made from those tires in large quantities.

RIVM, the Dutch National Institute for Public Health and the Environment, published a large report on artificial turf pitches in 2017 drawing on a survey of 100 pitches and conducted an extensive international literature review which details the results obtained in crumb rubber studies allowing international comparisons to be made [55]. It assessed human risks from such surfaces as “virtually negligible” and concluded the surfaces were safe to play on. A number of methodological and other questions have been raised about the report. The report itself, however, whilst noting chemical exposure limits in crumb rubber met EU commercial production standards added a caveat that some exceeded EU consumer products standards. They therefore proposed these PAH chemicals in pitches come closer to consumer standards and in a related report on play surface materials, they highlighted the surprising lack of knowledge about dose-response relationships of some chemicals when combined.

The European Chemicals Agency (ECHA) carried out a literature review on crumb rubber used on artificial pitches again in 2017 and its preliminary findings concluded that at most there was very low level about human exposures to the crumb [56]. However, again, important uncertainties were highlighted in the report’s conclusions and actions suggested. These included:

- Consider changes to the REACH Regulation to ensure rubber granules were only supplied with very low concentrations of PAHs and any other relevant hazardous substances.
- Ask owners and operators of existing (outdoor and indoor) fields to measure PAH and other substances’ concentrations in rubber granules used in their fields and making such information available to interested parties in an understandable manner.
- Ask producers of rubber granules and their interest organizations to develop guidance to help all manufacturers and importers of (recycled) rubber infill test their material.
- Ask European sports and football associations and clubs to work with the relevant producers to ensure information related to the safety of rubber granules in synthetic turfs is communicated in a manner understandable to the players and the general public.
- Have owners and operators of existing indoor fields with rubber granule infills ensure adequate ventilation.
- Recommend players using the synthetic pitches to take basic hygiene measures after playing on artificial turf containing recycled rubber granules.

A number of these recommendations had already been made by FIFA in 2015, although information is lacking as to the extent to which either football clubs or manufactures and suppliers complied with them or regulators ever checked on or used the suggestions themselves. The key FIFA guidance was that: “the manufacturer should be asked to supply to the purchaser an assurance that the sports surface together with its supporting layers, does not contain in its finished state any substance which is known to be toxic, mutagenic, teratogenic or carcinogenic when in contact with the skin. Furthermore that no such substances will be released as a vapor or dust during normal use” (Annex F4:26) [57].

Washington State in January 2017 published “a synthetic turf and cancer study”. The study found rates of cancer for soccer players reported were less than those expected given rates of cancer in Washington residents [58]. However, it recognized severe limitations in its own study and has been heavily critiqued in March 2017 by an epidemiologist and toxicologist who concluded the study was poorly designed and its conclusions not supported by its own data [59]. No other well designed and large epidemiological studies of sports players using 3G pitches currently exist.

The Toxics Use Reduction Institute in Massachusetts is currently working on an alternative assessment looking at the various options used for playing fields: natural grass, artificial turf with crumb rubber infill made from recycled tires, and artificial turf with other types of infill [60]. This should help to provide global purchasers of products, users and parents with more independent information on which to base their risk assessments. The industry itself has also developed alternative organic infills involving for example cork and coconut/coir materials instead of crumb rubber [61].

One other approach to tackling risk assessments of these rapidly changing technologies and materials may contribute to resolving the artificial turf dilemmas now emerging. This entails the adoption of the principles outlined in a WHO Charter on Environment and Health [62]. Hence, low impact technologies and good regulation are to be preferred. New technologies should only be introduced with prudence and not before appropriate prior assessment of potential environmental and health impacts and health data collection. Vulnerable and high risk groups including children should be especially protected. This would resonate with the recently published report in August 2016 from the UN Special Rapporteur dealing with human rights especially those of children and pregnant women to be protected from potential toxic pollutants [63]. Individuals should have a right to information and consultation on plans, decisions and activities that affect them and their health and those of their communities should take precedence over commercial interests. It would seem that many governments and organizations have failed at many levels to apply these basic principles in the past to the development of artificial pitches and playgrounds.

These approaches to crumb rubber, underpinned by the principles outlined above would be consistent with some recent developments and new research in setting standards for chemical carcinogens [64], creating “exposomes” that assess all pre-natal, post-natal and other exposures to environmental insults through a lifetime [65] and using cumulative health impact assessments to evaluate risk [66]. In July 2017, the Dutch Government also called for further restrictions on PAHs in crumb rubber used as synthetic turf infills [67]. This combines evidence-based and evidence-informed policy making with caution and may prove a wise choice.

## Figures and Tables

**Table 1:** Some key studies from the scientific literature indicating little potential risk from crumb rubber on 3G pitches and playgrounds for most but not all exposed groups and excluding reviews.

Subject	Type of Study/Methods/Size/Strengths and Weaknesses	Reference	Results
Environmental and health assessment of the use of elastomer granulates (virgin and from used tires) as filling in third-generation artificial pitches.	Lab-based using scenarios including looking at one pitch monitored over 11 months period and analysing VOC and metals including impacts on the wider environment and an evaluation of health risks from gas emissions. Total VOC for artificial turf was 8.3 μg/m^−3^ at 28 days. Relatively small study but one of the few to address risks to workers installing surfaces	[37]	VOCs and formaldehyde were either very or relatively low with levels highest and were not considered a health threat to sports people and spectators from crumb rubber. Largest risk was to workers laying turf over a 5 years period in small and poorly ventilated buildings.
Hydroxypyrene in urine football players after playing on artificial sports field with tire crumb infill.	Studied 7 non-smoking players aged 21 or more exposed for 2.5 h over 3 days period with intensive contact with crumb rubber. Lab-based plus only widely cited human biomonitoring study of crumb rubber chemicals. Very small study in scope, length and partly funded by industry. Controls for doses from other sources that may accumulate with crumb rubber chemicals	[41]	The researchers found no or minimal exposure to certain chemicals from urine samples and within range of uptake from environmental sources and/or diet. The total PAH of the pitch crumb rubber concentration was 24 mg/kg
Crumb-Rubber Infilled Synthetic Turf Athletic Fields. New York.	US study using artificial bio fluids looked at 8 artificial fibres, 8 infills and samples from 7 fields	[40]	Chemical exposures often below detection levels and metals at levels of “low” estimated human risks with the possible exception of lead.
Human Health Risk Assessment of Artificial Turf in Connecticut.	Air sampling from 1 indoor pitch with no active ventilation and 4 outdoor pitches in summer conditions. Testing for chemicals. Small study, limited range and researchers considered benzene levels recorded above background levels could have been due to personal sampling equipment problems. Ingestion potential not part of study	[38]	27 chemicals of concern at above background levels found and possibly field related in all 4 locations but the study concluded none were associated with elevated health risks. Benzothiazole on one pitch recorded at 6.5 μg/m^3^ on surface.
Artificial turf pitches—an assessment of health risks for football players. Oslo, Norway.	Exposure study based on 3 halls with granulates laid at different times and of different materials. Small study using scenarios of limited range. Study informed later inhalation and oral exposure studies.	[45]	Estimated that health risks were low VOC exposure inhalation Adults = 0.32 m^3^/kg body wt/day Juniors = 0.16 m^3^/kg body wt/day
An assessment of health risks for football players and the environment. National Institute of Public Health, Norway	Based on 3 reports. Weaknesses were that data on oral rubber intake were lacking, many VOCs monitored were not classified so health risks were unknown, many VOCs only had acute and not chronic effect classifications. It did note “artificial turf that contains substances of very high concern should not be used”	[36]	Estimated that health risks were low. Children may swallow some rubber granulate (1.0 g) during matches and/or training sessions Worst case inhalation measurements in Manglerudhallen (recycled rubber granulates), 234 different chemicals were found. This gave a total VOC of approximately 716 μg/m^3^ (indoor house VOC of 200 μg/m^3^)
Measurement of air pollution in indoor artificial turf halls	Measurements taken in a hall with recently laid rubber granulate (SBR rubber or Styrene Butadiene Rubber), a hall with rubber granulate (SBR rubber) which had been in use for one year and a hall with granulate made from thermoplastic elastomer. Noted gaps in literature on materials, some compounds not identified and some not studied.	[35]	Found low levels of many chemicals but noted “due to the dimensions of a football pitch, inadequate product research before launching a product will lead to a risk of undesirable exposure to chemicals with adverse health effects”
Evaluation of health risks of playing sports on synthetic turf pitches with rubber granulate	Literature review and analysis of rubber granulate from 100 Dutch pitches. 720 samples taken from 6 positions on each pitch. Limited number of migration tests used. Used artificial sweat and assessed migration of chemicals. Developed exposure scenarios for children including goalkeepers and adults. Recognition that the chemical exposures recorded in the study met general European limits for mixtures but would not meet the toy and consumer limit exposure limits. Assumes epidemiological studies reviewed are capable of assessing cancer risk but this is contested.	[55]	“Health risk of playing sports on synthetic turf pitches with an infill of rubber granulate is virtually Negligible”. Maximum pitch concentration found for BaP was 2.2 mg/kg dry matter Noted 2 earlier studies in Europe reported higher concentration of BaP than theirs with maximum values of 8.58 and 11 mg/kg. Low level of toluene, xylene and styrene detected but no benzene.

**Table 2:** Some studies/policy documents from the scientific literature indicating potential risk from crumb rubber on 3G pitches, commercial pavers and playgrounds.

Subject	Type of Study/Methods/Size/Strengths and Weaknesses	Reference	Results
Contents of metals and chemicals in artificial turf. Lab-based and exposure study	Recycled rubber granulate content from 13 Italian fields. Analysis of 25 metals, 9 PAHs. Air samples from 2 fields. Small sample size and lack of actual exposure scenarios issues.	[42]	BaP and zinc levels in granulate exceeded Italian “green” soil standards by 2 orders of magnitude. Worst case excess cancer risk assessment of BaP in air was 1 × 10^−6^. over 30 years at 0.4 ng/m^3^
Hazardous organic chemicals in rubber recycled tire playgrounds and pavers. Lab-based	21 rubber mulch samples collected from 9 different urban playgrounds and 7 commercial paver samples from a store. Analysis done for 31 PAHs, phthalates, anti-oxidants and of vapour phase above samples. Relatively small sample size, other sources of chemicals question raised.	[48]	High levels of several toxic chemicals found in the recycled materials especially BaP found in 5 samples. PAHs in commercial pavers was “extremely high”, up to 1%
PAHs, heavy metals release from rubber crumb in synthetic turf fields. Lab-based	Lab analysis of 9 football pitch samples of rubber crumb normally found in tires and their metals and PAHs. Risk assessment at 25 °C was done. Study preliminary and relatively small and acknowledges risk assessment over-estimates PAH contribution from fields.	[43]	Toxic equivalent of evaporates from crumb rubber “not negligible” and represented major part of total daily PAH intake by different routes. Noted “continuous release” of PAHs from evaporation and issue of chronic exposure
Threshold levels for carcinogens and policy implications of the NIOSH analysis	Review of chemical carcinogens policy. Looks at classification risk management limits and advocacy of a policy of elimination or substitution and implementation of engineering controls. Limitations include its purpose is to consider worker exposure although exposures to carcinogens do not differentiate between worker, consumer and citizen and the precautionary principle applies across the board.	[64]	“Underlying this policy is the recognition that there is no known safe level of exposure to a carcinogen and therefore that reduction of worker exposure to chemical carcinogens as much as possible through elimination or substitution and engineering controls is the primary way to prevent occupational cancer”

## Supplementary Materials

The following are available online at www.mdpi.com/1660-4601/14/9/1050/s1, Table S1: CRUMB Rubber Data Sheets Table—A Google search on June 10th produced about 12,400 references to crumb rubber MSDS without inverted commas. With inverted commas, it produced 215 results but not all related to specific MSDSs or for artificial turf and play surfaces, Table S2: HSE visits to crumb rubber/rubber granulate and artificial turf manufacturers & suppliers between 1 April 2005 and 31 March 2015.

## Abbreviations

CARACAL	the EU Competent Authorities for REACH and CLP
ECHA	European Chemical Agency
EU	European Union
ETRMA	European Tyre and Rubber Manufacturers Association
FIFA	Fédération Internationale de Football Association
HSE	UK Health and Safety Executive
KEMI	Swedish Chemicals Agency
PAHs	polycyclic aromatic hydrocarbons
REACH	European Registration, Evaluation, Authorisation and Restriction of Chemicals 2006
RIVM	Dutch National Institute for Public Health and the Environment
VOCs	volatile organic compounds
