# Supplementary material for: Artificial Turf: Contested Terrains for Precautionary Public Health with Particular Reference to Europe?
Source: Int J Environ Res Public Health. 2017 Sep 12;14(9):1050. doi: 10.3390/ijerph14091050 (PMC5615587; doi:10.3390/ijerph14091050)
Supplement: Supplementary file 1 [file ijerph-14-01050-s001.pdf]

<u>Table S1: CRUMB RUBBER DATA SHEETS TABLE</u> – A Google search on June 10th produced about 12,400 references to crumb rubber MSDS without inverted commas. With inverted commas, it produced 215 results but not all related to specific MSDSs or for artificial turf and play surfaces.

Manufacture supplier and date of MSDS	Data sheet product name and 'substance'	Toxicology information	Data gaps noted	Health effects listed	Occupational Exposure controls	Source of crumb
Charles Lawrence International July 2012	Recycled Tyre Rubber Granulate	"There is no data available for this product". (But ecological information is provided)<br><br>Other sections list information below. Not classified as dangerous according to the CHIP Regulations Chemicals (Hazard Information and Packaging for Supply Regulations 2002 - as amended).<br><br>Personal Protective Equipment:<br>Where engineering measures are not sufficient to maintain concentration of particulate below the relevant OEL's, suitable respiratory should be worn.<br>Eye:<br>Eye protection designed to protect against ingress should be worn as necessary.<br>Other:<br>Cotton/synthetic overalls or coveralls are normally suitable.	None	After Inhalation<br>Remove to fresh air.<br>After skin contact:<br>Wash skin thoroughly with soap and water and uses a proprietary skin cleaner.<br>* After eye contact:<br>Contact lenses should be removed. Irrigate copiously with clean, fresh water for at least 10 minutes holding the eyelids apart and seek medical advice.<br>After swallowing:<br>If accidentally swallowed obtain immediate attention.<br>Other:<br>In all cases of doubt or when symptoms persist, seek medical attention.	Inhalable dust: 8-hour TWA – 10mg/m².<br>Respirable Dust:<br>8-hour TWA – 5mg/m².<br>Type OES	
Allcocks 23/07/15	EPDM Crumb is an encapsulated blend of all the			May be irritating to the eye as a dust. May cause mild irritation only		The product in question is

	materials contained in EPDM rubber					ground, fine particle, vulcanised EPDM rubber. It has been recycled from an article.
Murfitts Data Sheet n/d	MiSport 2060	none	None	None	None	Recycled truck tyres
Recyclatech August 2012 (v5)	Microbially Devulcanised Rubber Crumb	TOXICOLOGY: No available data for this product. Dried material entities will be present derived from the biotechnology. This product is not expected to be classified as dangerous according to the Chemicals (Hazard Information & Packaging) Regulations. Adverse health effects are not expected if the product is handled in accordance with this Safety Data Sheet.logical process.		The product is classified as harmful Classification under the 1999/45/EC Dangerous Preparations Directive and amendments - but harm is aquatic . Promptly wash contaminated skin thoroughly with soap or mild detergent and water. Get medical attention promptly if any symptoms occur after washing.	INHALABLE DUST: 8-hour TWA - 10mg/m2. RESPIRABLE DUST: 8-hour TWA - 5mg/m2.Type OES. ENGINEERING MEASURES: Provide adequate ventilation. Where natural ventilation is inadequate, use of local exhaust ventilation and good general extraction may be necessary. PERSONAL PROTECTIVE EQUIPMENT: Where engineering measures are not sufficient to maintain concentration of particulate below the relevant OEL's, suitable respiratory protection	Surface Devulcanised Rubber Granulate from Waste Tyres

					should be worn.<br>EYE: Eye protection designed to protect against ingress should be worn as necessary.<br>OTHER:<br>Cotton/synthetic overalls or coveralls are normally suitable.	
Bri-chem<br>Nov 21 2013<br>Canada	Rubber crumb additive** for drilling fluid additive	Carcinogenicity<br>Untreated naphthenic/aromatic oils are classified as carcinogenic to humans by IARC. Some countries require the "R45" designation due to the presence of this oil.<br>Some rubber contains nitrosamines which have also been shown to be carcinogenic to animals in the laboratory. There is evidence that contact with ground rubber may aggravate pre-existing dermatitis		Slight skin irritant<br>Mechanical eye irritation.	ACGIH total dust TWA – 10mg/m^3<br>OSHA TWA mineral oil/distillates etc 15mg/m^3 Carbon Black 5mg/m^3<br>Also includes controls for zinc and sulphur.	Recycled tyres
Tyrec<br>Israel<br>April 12 2012	Ground Tire Rubber, Rubber crumb	Carcinogenicity<br>Untreated naphthenic/aromatic oils are classified as carcinogenic to humans by IARC. Some countries require the "R45" designation due to the presence of this oil.<br>Some rubber contains nitrosamines which have also been shown to be carcinogenic to animals in the lab.<br>There is evidence that contact	Embryotoxicity	Nuisance dusts can be irritating to the respiratory tract.	ACGIH total dust TWA – 10mg/m3<br>Carbon Black 3.5mg/m3<br>Also includes controls for zinc and sulphur	Recycled tyres

		with ground rubber may aggravate pre- existing dermatitis.				
Entech USA March 13 2009	Rubber crumb	Prolonged contact with untreated napthenic/aromatic oils caused skin cancer in mice when applied over a two year period. Untreated naphthenic/aromatic oils are classified as carcinogenic to humans by IARC. Some countries require the "R45" designation due to the presence of this oil. There is evidence that contact with ground rubber may aggravate pre- existing dermatitis. Some rubbers contain nitrosamines, which have also been shown to be carcinogenic to animals in the laboratory.			Reprocessed rubber Naphthenic/Aromatic Extender Oil, Carbon Black, Talc, Zinc oxide, Sulphur. TLVs etc as for other US MSDSs on crumb rubber	Recycled tyres
Perma Life USA 4/6/04	Tire Granules	No unusual hazard			Substrate is a mixture of natural and synthetic rubbers, carbon black and oils coating is a pigmented, polymerized dried film. Notes no established TLVs with OSHA or ACGIH	Reprocessed ground rubber
Delano USA 2016	" Green Clean" Crumb rubber	Rout of entry listed is only inhalation. Rubber not a carcinogen		Exposure signs and symptoms: Itching of skin, irritation of mucous membranes, sneezing and coughing, irritation of eyes.	Vulcanised rubber and talc listed. Notes ACGIH and OSHA PEL and TLV for vulcanised rubber not applicable and for talc it was 2mg/m^3	
Alllcock and	FKM crumb	Eye/face protection:	On long term	Occupational Exposure	Inhalable dust:	Recycled

Sons<br>UK<br>23/07/2015	Recycled filler	Wear safety glasses with side shields where there is a risk of dust generation that could lead to mechanical irritation of the eyes.<br>Hand protection: Protective gloves are not necessary, but recommended for normal use.<br>Body protection: Cotton or cotton/synthetic overalls or coveralls are normally suitable.	effects notes 'no information available'	Limits:	10mg/m3 (8hr TWA - OES)<br>Respirable dust: 5mg/m3 (8hr TWA - OES)	rubber
Liberty Tyre Recycling<br>USA<br>August 30th 2013	Rubber Mulch Feedstock. Natural rubber, synthetic rubber, carbon black, zinc oxide sulphur, filler, accelerators, anti-ozonants	Odor/vapors may be a nuisance in some individuals. In some individuals, short term exposure of material may produce mild and temporary discomfort to the respiratory tract resulting in wheezing, tightness in the chest, shortness of breath and coughing. Dust and small pieces of material may aggravate bronchitis, asthma, and emphysema if inhaled. Small cuts to the airway may result if pieces of metal are inhaled. Material contains small fibers, particulate matter and dust that may result in irritation (redness/itching) or other effects with some individuals. Small pieces of metal that protrude from some pieces of material may be able to create small cuts.<br>Eyes Material is abrasive if it enters the eye, which can cause irritation to sever damage if left untreated. Ingestion Irritation of mucus membranes of mouth,	Repro – no data available. Specific organ toxicity - no information available.	Carcinogenicity<br>This product contains a component (when isolated) that has been reported to be possibly carcinogenic (specifically when inhaled) based on its IARC,<br>ACGIH, NTP or EPA classification. Limited evidence to carcinogenicity in animal studies. IARC: 2B – Group 2B: Possibly carcinogenic to humans (Carbon black)		Recycled tyres

		throat, esophagus and stomach along with nausea may occur. Abrasion to the mouth, esophagus, stomach and intestinal tract may occur. Repeated exposure Repeated exposure to material may result in sensitization in susceptible individuals.				
Soft Surfaces Canada 30 June 2004	Soft Tile Rubber crumb and polyurethane	Rubber crumb from tire shreds is non-reactive under normal environmental conditions. The principal chemical component of tires is a blend of natural and synthetic rubber, but additional components include carbon black, sulfur, polymers, oil, paraffins, pigments, fabrics and bead or belt materials.		Potential Health Effects: Inhalation Products of combustion are irritating to the respiratory system.	This product is not regulated with respect to occupational exposure limits since the components are fully reacted products.	Recycled rubber tyres
Astlett Rubber, Ontario, Canada 23 September 2015	All natural rubber grades All Natural Rubber Grades (TSR, SMR, SIR, ADS, RSS, Crepes, DPNR, PA, SP, Hyflo)	Notes hydroxylamine is present but a t very levels : < 0.01		MSDS states 'natural rubber is not a hazardous material' and then lists acute and chronic effects as unknown and avoid ingestion and wear gloves.	None	

Table S2 HSE visits to crumb rubber/rubber granulate and artificial turf manufacturers & suppliers between 1 April 2005 and 31 March 2015					
**Year**	**Industry**	**Reactive?**	**Proactive?**	**Type**	**Details**
2009/10	Rubber crumb manufacture	Y	N	Investigation of Over 3 Day Injury	Accident on 04-10-09. Injured Person (IP) was using a large hook to clear a process blockage in an airveyor used to convey rubber crumb. Whilst pulling on the hook he felt a pain in his back. IP is a process operator in area which takes wet rubber crumb, dries it and then packages it into bales. He has been unable to carry out his normal range of duties since the accident and has been receiving physiotherapy. No Further Action (NFA).
2009/10	Tyre recycling	N	Y	Multi agency inspection following fire in rubber crumb	The Company operate three sites where waste tyres are processed. On 21st August a fire broke out at one site which destroyed the whole factory and a large quantity of whole and shredded tyres, and rubber crumb. The fire burnt for 8 weeks. This meeting was convened so that the HSE, EA and Fire Services could be satisfied that the measures to be put in place at another site would minimise the risk of a major fire, would incorporate lessons learnt from this site incident, and would take cogniscence of the current guidance and best practice available to tyre recyclers from the HSE, EA and other sources. The rubber residues, crumb and dust were collected by Local Exhaust Ventilation (LEV system and bagged for further recycling (at the site where the incident occurred). Also they have decided not to hold large stocks of tyres or shredded tyres at one of the other sites at all. The quantity to be stored will therefore be considerably less than that at the site where the fire occurred and the main product will be rubber crumb. NFA
2011/12	Tyre shredding to crumb	N	Y	Inspection - High Risk Sector*	Tyre shredding company. Prohibition Notice (PN) issued on tyre shredding equipment as not adequately safeguarded.

2012/13	Shreds and dyes rubber for use in surfaces	N	Y	Inspection after investigation	23/10/2012 - Inspection following investigation - Major Injury, Fractured Arm, 2 October 2012 - Left arm trapped between conveyor roller and conveyor belt causing injuries to arm. The company shreds and dyes rubber for use in surfaces. 30/10/2012 - Visit to site to advise on guarding of the shredding equipment. Written advice to make a Dangerous Substances and Explosive Atmospheres Regulations (DSEAR) risk assessment for storage of rubber crumb. Four improvement notices issued re access to dangerous parts of conveyors and no statutory examination of air receiver. Accident led to prosecution - £3,500 fine under Provision of Workplace Equipment (PUWER) Reg 11(1)
2013/14	Rubber crumb from old tyres	Y	N	Investigation of Over 3 Day Injury	IP worked cash in hand at site for dutyholder. Business idea was to shred old tyres and create "crumb" for sale onwards. Tyres were thrown into an initial shredder at one end of a series of conveyor belts and gradually ground down to rubber and steel (from sidewalls) for bagging. No proper guarding on machinery or isolation devices or accessible emergency stops. IP was trying to clear a potential blockage of one of the belts caused by build-up of rubber crumb with a trowel when the trowel and quickly afterwards his arm were caught and pulled around a roller on the belt. Accident not reported. HSE made aware on 24 September 2014 by solicitor approached by IP for civil claim. Visited site on 24/09 - work no longer going on and company in liquidation. NFA
2013/14	Artificial turf manufacture	N	Y	Inspection - High Risk Sector*	05 September 2013. Site Visit Artificial Turf manufacture on (old) carpet making machines. Process involves adding a latex backing to a woven mat and then stitching artificial grass threads through the mat. Two Material Breaches identified: Insufficient safeguarding at the in-running nip on the Singer machine and no suitable safe system of work at the stitching point. Notice of Contravention (NoC) issued. (Company are already addressing both machinery safety issues so no Improvement Notices issued).

2013/14	Rubber recycling for playground surface material	N	Y	Inspection - Priority Local Inspection	Joint Visit (JV) to site with Inspector on 10 September. General recycling of rubber products, mostly tyres etc. Product is mostly used as playground surface material/garden use. NoC and PN left at site covering work at height (PN issued), vehicle maintenance and general guarding of slow moving conveyors.
2013/14	Retread or crumbing of tyres	N	Y	Inspection - Priority Local Inspection	1/5/13 Company retread or crumb tyres. Large contract recently obtained requiring company to increase size of crumb storage piles above the 3 metre max height recommended in HSE guidance. Company have recognised the increased potential for spontaneous combustion and increase in crumb storage agreed and under regular inspection from Environmental Agency (EA). NOC issued re material breach Reg 3 MHSW regs. Improvement Notice served - a suitable & sufficient risk assessment of the risks to safety of the employees when operating & maintaining the tyre presses on site, has not been made.
2014/15	Artificial turf manufacture	N	Y	Inspection of machinery guarding as part of intervention	Ongoing intervention with Company. The outstanding issue is safeguarding on their artificial turf making machines, specifically at the tufting heads (the bit on a carpet making machine where the thread gets stitched in to the carpet). This is a matter of concern as the tufting heads are capable of drawing a victim in to the machine (and stitching them to the carpet in the process) Sector to look at the industry wide standards of safeguarding on tufting machines.

2014/15	Tyre recycling - on site crumber	N	Y	Inspection - High Risk Sector*	Tyre recycling company, recently started up and not yet processing. Temp licence to store and recycle tyres, in process of establishing business. Lot of stock coming in, but bought crumber with burnt out starter on motors, needs to get new one made. Initial complaint received - matters raised were no Fork Lift Truck (FLT) certificate, unguarded machinery. Product to be bagged and transported off site for manufacture into other products such as insulation, tarmac, water control materials using new bonding material. Feed for crumber would give concern as it is currently a sloping ramp from a mezzanine. No letter but revisit to be made in due course when producing. Follow-up in June 2015 - Only work activity ongoing at the moment is storage of lots of mixed tyres, with no processing of any sort happening or planned, as crumber is now going.

Notes

1. Crumb rubber and rubber granulate manufacturers (and those using recycled tyres) are not separately identified by Standard Industrial Classification (SIC) – they are part of SIC 221 – Manufacture of rubber products.
2. Given point 1, investigation and inspection records for SIC 221 were extracted and then analysed to identify where key words such as ‘crumb’, ‘tyre’ , ‘recycle’ were used in the Inspector notes.
3. A further search of investigation and inspection records across all industry was carried out using key words such as ‘crumb’ to identify relevant companies.
4. Artificial turf manufacturers are not separately identified by Standard Industrial Classification (SIC)
5. Given point 4, investigation and inspection records for Artificial turf manufacturers, etc. were identified by analysing all investigations and inspections and using key words such as ‘artificial’, ‘turf’ , ‘leachate’, etc. to identify relevant records.
6. Given the above process, the records found cannot be described as definitive.
7. *These 3 inspections have been incorrectly coded on our database as ‘high risk sector’.
